# Inflammatory markers predict survival in patients with postoperative urothelial carcinoma receiving tislelizumab (PD-1 inhibitor) adjuvant therapy

**DOI:** 10.1186/s12885-024-11969-5

**Published:** 2024-02-12

**Authors:** Meng Yang, Jingwen Zhang, Dongqun Wei, Tianyi Yu, Zeyu Chen, Xin Liu, Haitao Zhu

**Affiliations:** Department of Urology, The Affiliated Hospital of Xuzhou Medical University, Xuzhou, China

**Keywords:** Immunotherapy, Urothelial carcinoma, Inflammatory markers, Tislelizumab, Survival

## Abstract

**Background:**

In the management of urothelial carcinoma, patient selection for immunotherapy, particularly with immune checkpoint inhibitors such as PD-1 (programmed cell death protein 1), is important for treatment efficacy. Inflammatory markers are useful for predicting treatment outcomes and immune-related adverse events (irAEs). This study aims to retrospectively explore the associations between inflammatory markers and outcomes in patients with postoperative urothelial carcinoma undergoing tislelizumab (PD-1 inhibitor) adjuvant therapy.

**Methods:**

A retrospective analysis was conducted on 133 patients with postoperative urothelial carcinoma who received tislelizumab adjuvant therapy at the Affiliated Hospital of Xuzhou Medical University from April 2020 to August 2023. The prognostic effects of the neutrophil-to-lymphocyte ratio (NLR), platelet-to-lymphocyte ratio (PLR), and monocyte-to-lymphocyte ratio (MLR) on disease-free survival (DFS) and overall survival (OS) were assessed using Cox regression models. The correlation between inflammatory markers and the onset of irAEs was analyzed using logistic regression models.

**Results:**

NLR < 5 and MLR < 0.31 were significantly associated with better outcomes compared to NLR >5 and MLR >0.31, respectively. Multivariate analysis revealed that an NLR < 5 was independently associated with better DFS and OS. However, there was no significant effect on the DFS and OS between PLR < 135 and PLR >135. Patients who experienced irAEs had longer DFS and OS. Multivariate analysis demonstrated that irAEs were an independent prognostic risk factor for DFS and OS. There was no significant difference in the occurrence of irAEs among different NLR, PLR, and MLR groups.

**Conclusion:**

In patients with postoperative urothelial carcinoma receiving tislelizumab adjuvant therapy, the assessment of NLR and MLR before treatment may serve as valuable predictive markers of clinical outcome.

**Supplementary Information:**

The online version contains supplementary material available at 10.1186/s12885-024-11969-5.

## Introduction

The incidence of urothelial carcinoma (UC) ranks seventh among all tumors and fourth among men, among which bladder cancer accounts for 90–95% of the cases and upper urothelial carcinoma accounts for 5–10% [1]. The standard treatment for patients with muscle-invasive urothelial carcinoma is radical surgery, which includes cystectomy for tumors originating in the bladder and nephroureterectomy for tumors originating in the upper urinary tract [2]. However, more than half of the patients with pathological confirmation of cancer invading the muscularis propria or affecting the nearby lymph nodes will experience fatal metastatic recurrence [3, 4]. Adjuvant chemotherapy may prolong disease-free survival (DFS) in patients with locally advanced upper tract urothelial carcinoma (UTUC) [5–7]. However, there is no consensus on the routine use of adjuvant cisplatin-based chemotherapy. A meta-analysis by NECCHI et al. included 1554 patients from 15 centers, and the results indicated that adjuvant chemotherapy failed to improve the overall survival (OS) rates for patients with UTUC [8].

In recent years, multiple immune checkpoint inhibitors have been approved for the treatment of urothelial cancer [9–11]. The immune system plays an important role in monitoring and clearing mutant cells. Under the protection of the tumor microenvironment, tumor cells are not found, recognized and killed by the immune system. The reason may be that tumor cells release antigens into the blood, antigen-presenting cells present antigens on the surface of tumor cells to T cells, and T cells are activated and transported and infiltrated around the tumor to recognize and kill tumor cells. PD-1 (Programmed Cell Death Protein-1) /PD-L1(Programmed Cell Death Ligand-1) can negatively regulate immune function and prevent immune self-killing, while tumor cells can cleverly escape the killing effect of T cells [12]. PD-1 is mainly expressed on activated CD4+ T cells, CD8+ T cells, natural killer cells, B cells and activated monocytes, and its ligand PD-L1 is widely expressed on T cells, B cells, dendritic cells, macrophages and other tissues. When the two combine, they inhibit immune regulation and T cell surveillance, so that T cells are inactivated and tumor cells escape from immune surveillance and killing [13]. Tislelizumab can specifically bind to PD-1, block the interaction between PD-1 and its ligand (PD-L1) and terminate the PD-1 immunosuppressive signal caused by the interaction of PD-1 and PD-L1 in T cells, which enables T cells to resume the immune response against tumors [14]. However, immunotherapy does not benefit every patient with cancer. Currently, there have been no reliable predictive markers to identify patients who are most likely to benefit from a particular therapy [15]. Hence, the identification of valuable and dependable predictive markers suitable for regular clinical use is of paramount importance. The discovery of such markers, particularly inflammatory markers, could lead to a practically noninvasive and clinically convenient test.

Inflammation is associated with tumorigenesis and tumor progression, as it facilitates a conducive environment that supports cancer cell growth and spread and also activates carcinogenic signaling pathways. Therefore, inflammatory factors have the potential to function as biomarkers for predicting tumor recurrence and patient prognosis. Previous studies have shown that the neutrophil-to-lymphocyte ratio (NLR), platelet-to-lymphocyte ratio (PLR), and monocyte-to-lymphocyte ratio (MLR) are used as markers to predict survival in some malignant tumors [16–18]. We examined whether these inflammatory markers have prognostic value in patients with postoperative urothelial carcinoma receiving tislelizumab adjuvant therapy. The study focused on the correlation between NLR, MLR and PLR and DFS as the primary endpoint, and OS and immune-related adverse events (irAEs) as secondary endpoints.

Some patients continue to experience adverse events despite their improved tolerability to immune checkpoint inhibitors compared to conventional chemotherapy. This study explored the associations between inflammatory markers (NLR, PLR, and MLR) and the occurrence of irAEs in patients with postoperative urothelial carcinoma undergoing tislelizumab adjuvant therapy.

### Patients and methods

Patients with postoperative urothelial carcinoma who received tislelizumab adjuvant therapy at the Affiliated Hospital of Xuzhou Medical University from April 2020 to August 2023 were selected for this retrospective study. Patients’data including their individual NLRs, PLRs, and MLRs were collected from electronic medical records. These ratios were calculated using the latest hematological data, obtained within 3 weeks before receiving tislelizumab adjuvant therapy. We de-identified all patient details. Written informed consent was obtained from the patients involved, and the study design was approved by the research ethics committee of the above institution. The last date of follow-up was in January 2024.

The inclusion criteria for the study were as follows: (1) patients who underwent radical cystectomy or radical nephroureterectomy following a confirmed pathological diagnosis of urothelial carcinoma; and (2) patients who received a minimum of two drug infusions and underwent pre-treatment peripheral blood testing. The exclusion criteria were as follows: (1) patients who had received alternative antitumor therapies before receiving anti-PD-1 therapy; (2) patients with other tumors; (3) patients with infectious condition before receiving anti-PD-1 therapy; and (4) those with incomplete data or those lost to follow-up.

Tislelizumab was administered every 3 weeks at a dose of 200 mg. Data on age, gender, smoking history, body mass index, T stage, N stage, occurrence of adverse events, baseline biochemical parameters (before the first treatment cycle), any concomitant treatments, and treatment responses of the patients were collected retrospectively.

Every 3 months, scheduled computed tomography or magnetic resonance imaging was conducted to assess the response to treatment. DFS was calculated from the start of postoperative immune adjuvant therapy to the date of disease progression, whereas OS was measured from the date of initiation of immunotherapy to the last contact or date of death. The reporting of this study conforms to the STROBE guidelines [19].

### Statistical analysis

Patients were stratified into low-NLR and high-NLR (< 5 and>5), low-PLR and high-PLR (< 135 and>135), or low-MLR and high-MLR (< 0.31 and>0.31) groups based on previously established cutoff values [15, 20]. Kaplan–Meier methodology was utilized to construct survival curves for DFS and OS. The log-rank test was employed to evaluate disparities between various groups. Cox regression models were utilized to identify independent prognostic indicators linked to DFS and OS. Factors identified to be statistically significant in the univariate analysis were included in the multivariate analysis. Logistic regression analysis was used to investigate the correlation between inflammatory markers and the occurrence of irAEs. A *P*-value < 0.05 was considered statistically significant, and all statistical analyses were conducted using SPSS version 26 (IBM, Armonk, NY, USA).

## Results

**Fig. 1:**
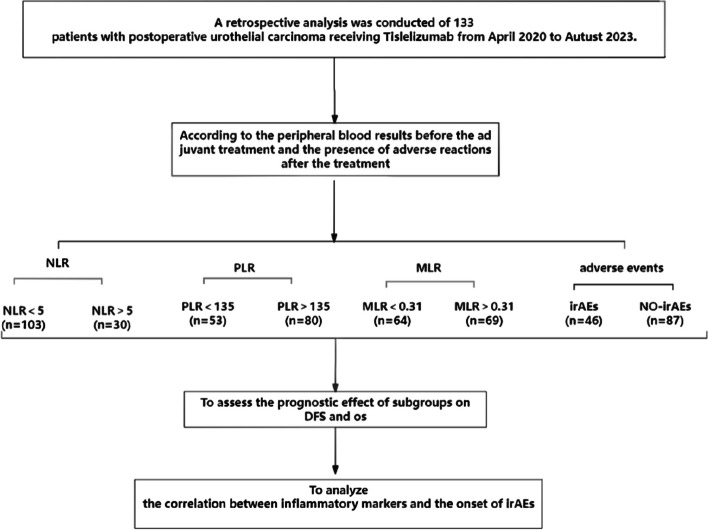
Flowchart of the study

The clinical characteristics of the patients are summarized in Table 1. A total of 133 patients with postoperative urothelial carcinoma receiving Tislelizumab adjuvant therapy were participated in this study, including 38 female (28.6%) and 95 male (71.4%). Of these, 53 (39.8%) were diagnosed with bladder cancer and 80 (60.2%) were diagnosed with UTUC. No differences were observed between any cancer type and the various inflammatory marker groups. Similarly, no differences were observed between any inflammatory marker and concomitant treatments, including chemotherapy. Figure 1 demonstrates the flowchart of the study.

**Table 1:** Characteristics of patients in this study

Characteristic	Total (*n=*133) N(%)	NLR			PLR			MLR		
		<5 (*n=*103, 77.4%)	>5 (*n=*30, 22.6%)	*p*	<135 (*n=*53, 39.8%)	>135 (*n=*80, 60.2%)	*p*	<0.31 (*n=*64, 48.1%)	>0.31 (*n=*69, 51.9%)	*p*
**Sex**				0.845			0.106			0.113
Female	38(28.6%)	29	9		11	27		24	14	
Male	95(71.4%)	74	21		42	53		40	55	
**Age**	64.6±11.0	64.1±10.7	66.2±11.1	0.342	63.8±11.5	65.1±10.4	0.538	63.2±10.8	65.9±11.4	0.144
**BMI**	23.4±3.4	23.3±3.4	23.6±3.4	0.744	23.2±3.7	23.5±3.2	0.657	23.8±3.5	23.0±3.3	0.179
**Smoke**				0.311			0.052			0.223
Yes	90(67.7%)	72	18		41	49		40	50	
No	43(32.3%)	31	12		12	31		24	19	
**Cancer type**				0.213			0.753			0.063
UTUC	80(60.2%)	59	21		31	49		29	51	
BC	53(39.8%)	44	9		22	31		35	18	
**pT stage**				0.916			0.366			0.512
pT2	75(56.4%)	62	13		30	45		45	30	
pT3	44(33.1%)	32	12		18	26		15	29	
pT4	14(10.5%)	9	5		5	9		4	10	
**Lymph node involvement**				0.079			0.858			0.071
pN0	119(89.5%)	92	27		49	70		55	59	
pN+	14(10.5%)	11	3		4	10		9	10	
**With chemotherapy**				0.244			0.382			0.213
Yes	108(81.2%)	79	29		43	65		45	63	
No	25(18.8%)	20	5		10	15		19	6	
**Adverse events**				0.482			0.108			0.077
No	87(65.4%)	69	18		39	48		37	50	
Yes	46(34.6%)	34	12		14	32		27	19	

*pN0* no lymph node involvement, *pN+* lymph node involvement

**Fig. 2:**
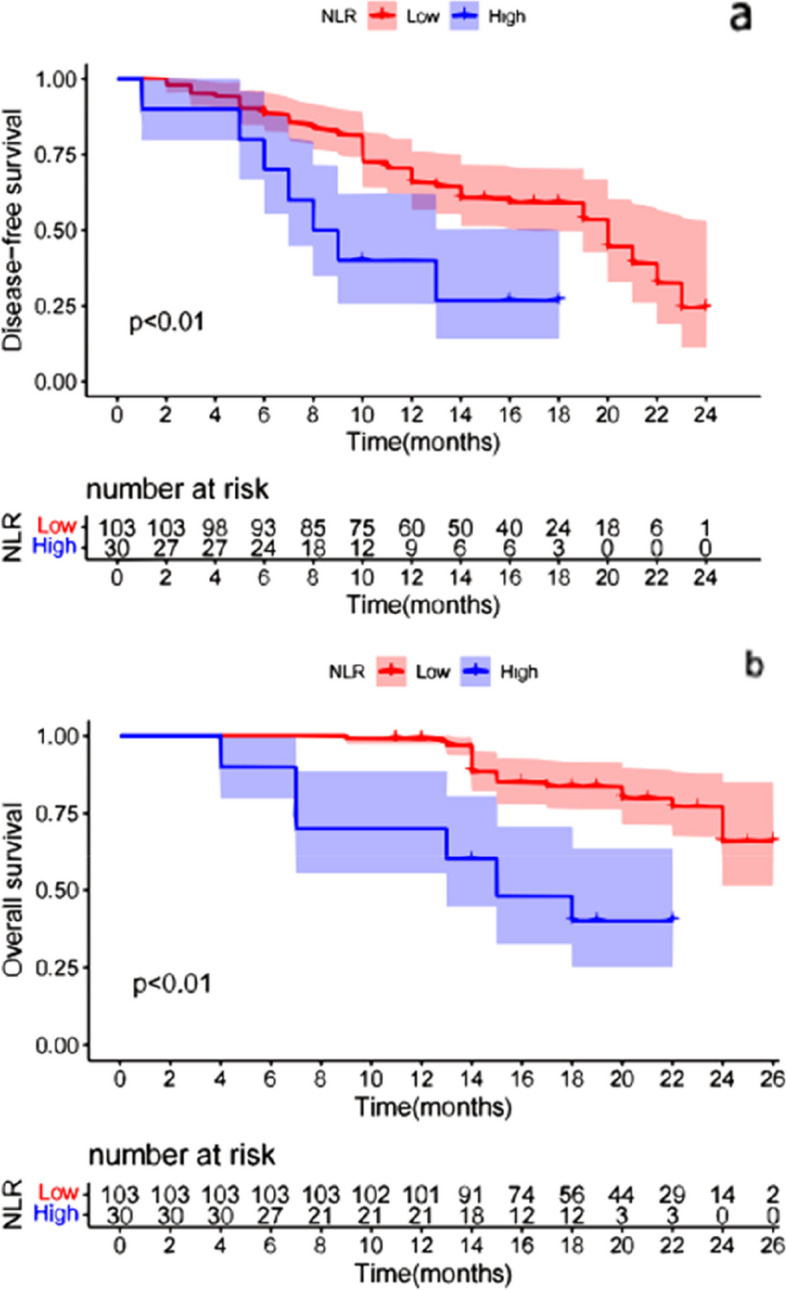
Comparison of DFS(a) and OS(b) curves between patients with different groups (NLR**<**5, NLR**>**5). **a**: Patients with low NLR had longer DFS [HR 2.740 (95% CI, 1.008, 3.680); *p* < 0.01]; **b**: Patients with low NLR had longer OS [HR 4.817 (95% CI, 1.572, 4.600); *p* < 0.01]

**Fig. 3:**
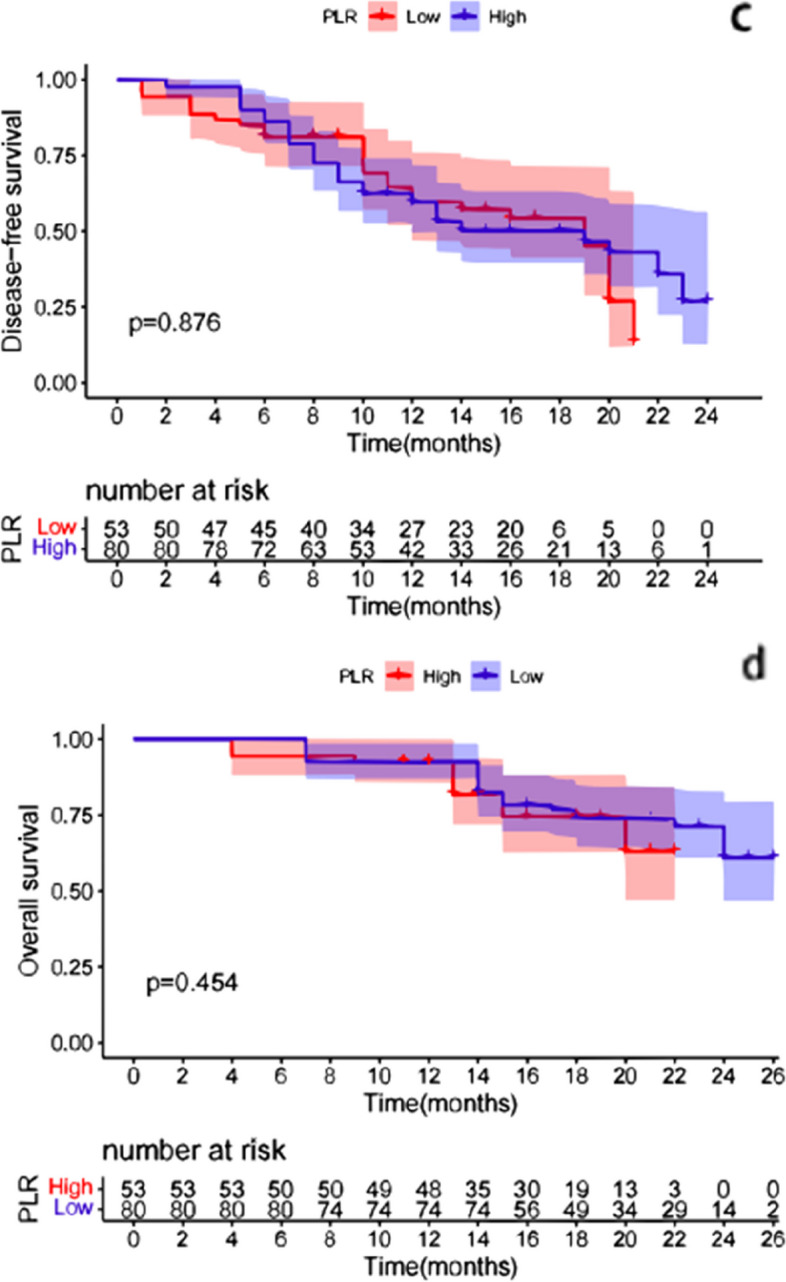
Comparison of DFS(c) and OS(d) curves between patients with different groups (PLR**<**135, NLR**>**135). **c**: There were no significant differences in DFS between the high PLR group and the low PLR group [HR 0.962 (95% CI, 0.038, 0.150); *p* = 0.876]; **d**: There were no significant differences in OS between the high PLR group and the low PLR group [HR 0.778 (95% CI,0.251, 0.721); *p* = 0.454]

**Fig. 4:**
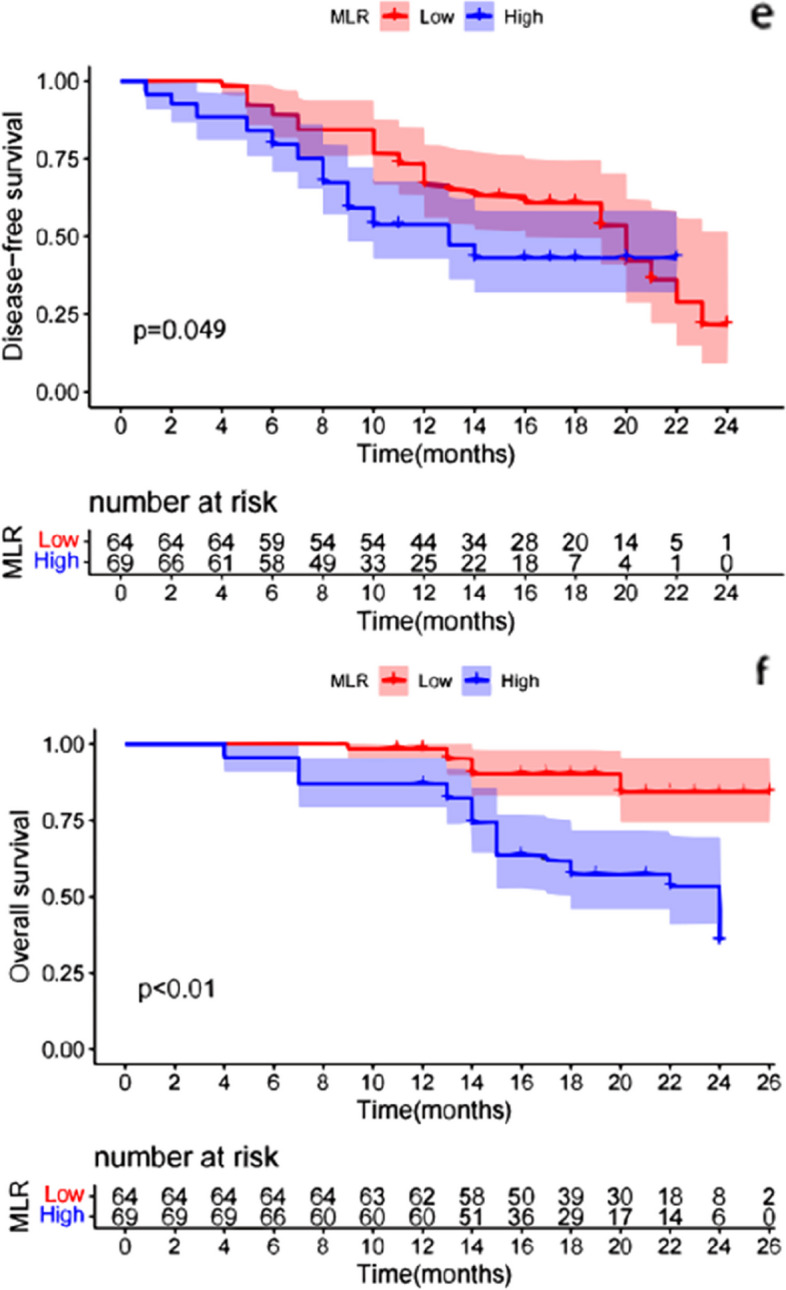
Comparison of DFS(e) and OS(f) curves between patients with different groups (MLR**<**0.31, MLR**>**0.31). **e**: Patients with low MLR had longer DFS [HR 1.067 (95% CI, 0.474, 1.884); *p* = 0.049]; **f**: Patients with low MLR had longer OS [HR 4.289(95% CI, 1.456, 3.638); *p* < 0.01]

**Fig. 5:**
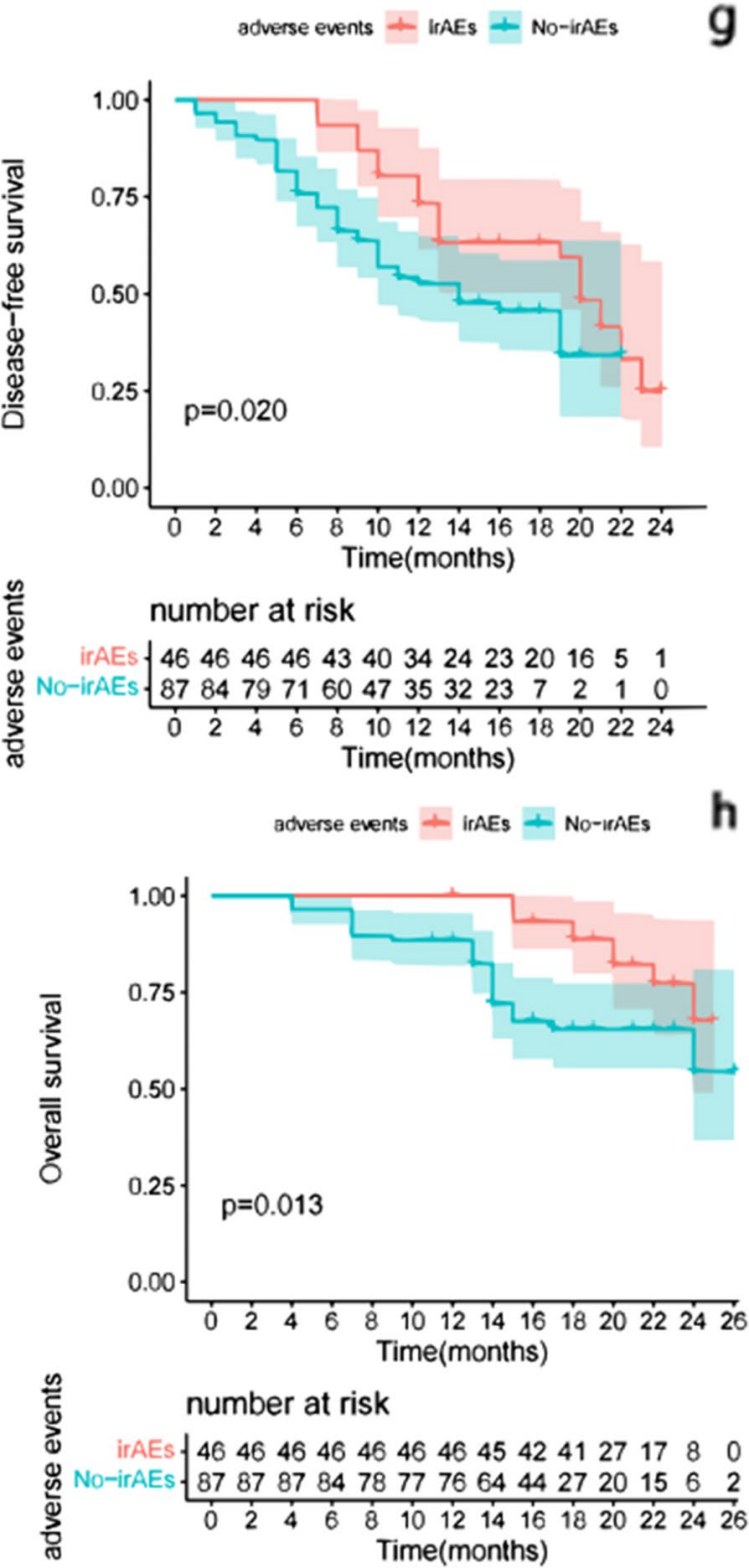
Comparison of DFS(g) and OS(h) curves between patients with different groups (irAEs, No-irAEs). **g**: Patients with irAEs had longer DFS [HR 1.872 (95% CI, 0.627, 2.275); *p* = 0.020]; **f**: Patients with irAEs had longer OS [HR 2.548(95% CI, 0.935, 2.415); *p* = 0.013]

The K-M survival curve was used to analyze the differences in patient survival outcomes, and the results showed that the patients with low NLR had longer DFS [HR 2.740 (95% CI, 1.008, 3.680); *p* < 0.01]; Patients with low NLR had longer OS [HR 4.817 (95% CI, 1.572, 4.600); *p* < 0.01] (Fig. 2). There were no significant differences in DFS between the high PLR group and the low PLR group [HR 0.962 (95% CI, 0.038, 0.150); *p* = 0.876]. There were no significant differences in OS between the high PLR group and the low PLR group [HR 0.778 (95% CI,0.251, 0.721); *p* = 0.454] (Fig. 3). Furthermore, patients with low MLR had longer DFS [HR 1.067 (95% CI, 0.474, 1.884); *p* = 0.049] and patients with low MLR had longer OS [HR 4.289(95% CI, 1.456, 3.638); *p* < 0.01] (Fig. 4). Finally, we found that the patients with irAEs had longer DFS [HR 1.872 (95% CI, 0.627, 2.275); *p* = 0.020] and patients with irAEs had longer OS [HR 2.548(95% CI, 0.935, 2.415); *p* = 0.013] (Fig. 5).

Univariate Cox regression analysis was performed for all variables, and those with a *P* < 0.05 was included in multivariate Cox regression analysis. Univariate analysis revealed that the NLR exhibited a significantly associated with DFS and OS (Table 2). Similarly, the MLR group exhibited a significantly associated with DFS and OS. But, multivariate analysis demonstrated that MLR was not an independent prognostic risk factor for DFS and OS. There were no significant differences in DFS between the high-PLR group and the low-PLR group. Furthermore, the pT4 stage and irAEs exhibited a significantly associated with DFS and OS. Multivariate analysis revealed that NLR, pT stage and irAEs were significantly associated with DFS and OS (Tables 2 and 3). Multivariate analysis demonstrated that NLR, pT stage and irAEs were independent prognostic risk factor for DFS and OS.

**Table 2:** Univariate and multivariate analyses of DFS

Variables	Disease-free survival			
	Univariate analyses	*p*	Multivariate analyses	*p*
	Hazara ratios		Hazara ratios	
	(95%CI)		(95%CI)	
Sex	0.938(0.556–1.583)	0.811		
Age	1.003(0.980–1.027)	0.804		
BMI	0.986(0.917–1.060)	0.698		
Smoke	1.007(0.605–1.676)	0.978		
pT stage				
pT2	Ref.			
pT3	2.017(1.156–3.520)	0.014	1.683(0.906–2.810)	0.155
pT4	6.303(3.066–12.956)	< 0.001	7.536(3.131–11.235)	< 0.001
NLR	0.365(0.213–0.625)	< 0.001	0.282(0.138–0.575)	< 0.001
PLR	1.030(0.624–1.700)	0.908		
MLR	0.607(0.371–0.995)	0.049	1.152(0.604–2.196)	0.668
Chemotherapy	1.597(0.835–3.056)	0.157		
Adverse events	0.541(0.315–0.929)	0.026	0.444(0.254–0.776)	0.004

*CI* Confidence interval, *NLR* Neutrophil-to-lymphocyte ratio, *PLR* Platelet-to-lymphocyte ratio, *MLR* Monocyte-to-lymphocyte ratio, *irAEs* Immune-related adverse events

**Table 3:** Univariate and multivariate analyses of OS

Variables	Overall survival			
	Univariate analyses	*p*	Multivariate analyses	*p*
	Hazara ratios		Hazara ratios	
	(95%CI)		(95%CI)	
Sex	1.067(0.526–2.162)	0.858		
Age	1.003(0.971–1.035)	0.877		
BMI	0.989(0.896–1.093)	0.833		
Smoke	0.877(0.451–1.708)	0.700		
pT stage				
pT2	Ref.			
pT3	1.850(1.070–3.197)	0.028	1.382(0.736–2.167)	0.038
pT4	2.704(1.363–5.364)	0.004	1.945(0.866–3.879)	0.019
NLR	0.215(0.110–0.420)	< 0.001	0.206(0.090–0.472)	< 0.001
PLR	1.290(0.652–2.552)	0.464		
MLR	0.240(0.109–0.526)	< 0.001	0.509(0.206–1.260)	0.144
Chemotherapy	1.450(0.604–3.482)	0.406		
Adverse events	0.400(0.187–0.855)	0.018	0.268(0.118–0.610)	0.002

*CI* Confidence interval, *NLR* Neutrophil-to-lymphocyte ratio, *PLR* Platelet-to-lymphocyte ratio, *MLR* Monocyte-to-lymphocyte ratio, *irAEs* Immune-related adverse events

Grade I or II irAEs accounted for the majority of irAEs observed in our study. A total of 46(34.6%) patients had irAEs, including 18 (39.1%) with constipation, 11 (23.9%) with skin-related issues, 9 (19.7%) with diarrhea, 5(10.9%) with pyrexia, and 3 (6.5%) with hypothyroidism. The median DFS of the 87 patients who did not experience irAEs was significantly shorter compared to the 46 patients who experienced irAEs (Table 2 and Fig. 5). Multivariate analysis revealed that the presence of irAEs was an independent prognostic risk factor for DFS and OS (Tables 2 and 3).

The incidence of irAEs was not significantly associated with NLR, PLR, or MLR. In the low NLR group (< 5), the rate of irAEs was 33.0%, whereas in the high NLR group (> 5), it was 40.0% (*P* = 0.480). Similarly, for the low PLR group (< 135), the rate of irAEs was 26.4%, whereas in the high PLR group (> 135), it was 40.0% (*P* = 0.109). In the low MLR group (< 0.31), the rate of irAEs was 42.2%, whereas in the high MLR group (> 0.31), it was 27.5% (*P* = 0.078; Table 4).

**Table 4:** Levels of the peripheral blood markers by irAEs development

Blood parameter	irAEs, n(%)	Univariate		Multivariate	
		OR(95%CI)	*P*	OR(95%CI)	*P*
NLR<5(*n=*103)	34(33.00)	0.739(0.320-1.79)	0.480	0.383(0.125-1.178)	0.094
NLR>5(*n=*30)	12(40.00)	1		1	
PLR<135(*n=*53)	14(26.42)	0.538(0.253-1.148)	0.109	0.605(0.274-1.338)	0.215
PLR>135(*n=*80)	32(40.00)	1		1	
MLR<0.31(*n=*64)	27(42.19)	1.920(0.930-3.964)	0.078	1.138(0.501-1.583)	0.059
MLR>0.31(*s*69)	19(27.54)	1		1	

*OR* Odds ratio, *CI* Confidence interval, *NLR* Neutrophil-to-lymphocyte ratio, *PLR* Platelet-to-lymphocyte ratio, *MLR* Monocyte-to-lymphocyte ratio, *irAEs* Immune-related adverse events

## Discussion

Comprehensive treatment with radical cystectomy remains the standard treatment for muscle invasive urothelial carcinoma [21]. The advent of immunotherapy has brought hope for patients with postoperative urothelial carcinoma. Tislelizumab, in particular, has shown significant clinical advantages and a manageable safety profile [14]. Despite these advantages, many clinical studies have shown that only some patients benefit from this treatment. Hence, there is an urgent need for effective inflammatory markers to identify individuals who are most likely to respond positively to such therapies.

In cancer, the dysregulation of the PD-1/PD-L1 axis enables cancer cells to evade the immune system, and the overexpression of PD-L1 is associated with poor prognosis in patients with melanoma, lung, and ovarian cancers [13, 22]. For patients with urothelial carcinoma, particularly those who have experienced disease progression during or after platinum-based chemotherapy, antibodies targeting PD-1/PD-L1 have emerged as viable first-line treatment alternatives.

At present, the PD-L1 level serves as a widely utilized marker to predict the effectiveness of immunotherapy. A randomized controlled clinical trial called Checkmate274 showed that the adjuvant treatment experimental group, with a maximum treatment duration of 12 months, showed superior efficacy compared to the placebo. The median DFS for nivolumab was 20.8 months, considerably longer than the 10.8 months observed in the placebo group. The median OS for nivolumab was 22.9 months, in contrast to 13.7 months for the placebo group. Patients with PD-L1 expression ≥1% experienced a more significant benefit in DFS. Subgroup analyses showed that DFS was superior with nivolumab compared to placebo across different levels of PD-L1 expression and irrespective of prior neoadjuvant cisplatin chemotherapy [2]. While tumor mutational burden and microsatellite instability-high are also emerging as predictive markers, their detection technologies are currently underdeveloped and expensive, limiting their large-scale clinical applications [15]. Conversely, the identification of inflammatory markers could offer a clinically convenient and practically noninvasive testing method.

The activation of carcinogenic signaling pathways and the promotion of a conducive microenvironment for the growth and metastasis of cancer cells are closely associated with inflammation [23]. The inflammation status is well reflected by the markers (NLR and MLR) evaluated in this study. Our findings underscore the prognostic significance of NLR in urothelial carcinoma. Additionally, we revealed a previously unreported association between NLR, MLR, PLR, and irAEs in patients with urothelial carcinoma undergoing tislelizumab adjuvant therapy. In previous studies, the prognostic significance of NLR has been established in patients with cancer receiving immunotherapy for late-stage melanoma or non-small-cell lung cancer [24]. For example, in a retrospective study of 187 patients with metastatic melanoma treated with ipilimumab, a favorable association with improved outcomes was observed in those with NLR < 5 [25]. Consistent with these findings, our study found that NLR <5 was significantly associated with DFS and OS. Comprehensive treatment with radical cystectomy remains the standard treatment for muscle invasive urothelial carcinoma.

In addition, our findings suggest that MLR>0.31 was associated with worse DFS and OS. In patients with metastatic gastric and colorectal cancers, previous research has established the reliability and independence of MLR as a laboratory biomarker that is readily applicable for predicting clinical outcomes [20, 26]. In the current study, the univariate analysis shows that MLR is only a factor influencing DFS, but in the multivariate analysis, it does not prove a significant prognostic impact. It is likely that the level of MLR value or the cutoff value may vary depending on the specific inflammatory condition due to variations in tumor type and stage. High PLR has been reported to be linked with unfavorable outcomes and serves as a valuable predictor of the efficacy of anti-PD-1 therapy in many cancers [27, 28]. However, our study concluded that PLR >135 was not associated with worse DFS and OS.

IRAEs, also known as dysimmune toxicities, can be induced by immunotherapies and predominantly affect the gut, skin, endocrine glands, liver, and lung, although they have the potential to affect any tissue [29]. However, our study revealed that patients who experienced irAEs had longer DFS and OS. Similarly, in a previous study, patients who experienced irAEs had better PFS [30]. Zhang et al. reported that patients with melanoma treated with nivolumab who experienced irAEs had better OS [31]. In our study, we also explored the potential link between irAEs and the peripheral blood markers NLR, PLR, and MLR. However, our findings revealed no significant association between these markers and the occurrence of irAEs.

Apart from the small sample size, the limitations of the present study include its retrospective design and data collection at a single institution, potentially reducing the study’s power and weakening the predictive capacity of inflammatory markers. In addition, the median OS could not be calculated because of the short follow-up period. Further validation through randomized studies with an untreated control group is needed to confirm the predictive significance of inflammatory markers on DFS, OS, or irAEs. Moreover, the PD-L1 status was known in so few patients that we could not include it in our study. There remains ambiguity regarding the correlation between inflammatory marker levels and PD-L1 expression. Last but not least, the undetermined optimal cutoff values for these biomarkers may introduce potential bias and heterogeneity due to the different cutoff thresholds. Despite these limitations, the integration of the NLR, PLR, and MLR with clinicopathological factors and other prognostic indicators may assist clinicians in risk stratification. This could help tailor treatment strategies and improve outcome predictions for patients with postoperative urothelial carcinoma receiving tislelizumab (PD-1 inhibitor) adjuvant therapy.

## Conclusion

In summary, the pretreatment inflammatory markers examined in this study, namely NLR and MLR, may be correlated with outcomes in patients with postoperative urothelial carcinoma undergoing tislelizumab adjuvant therapy. These findings offer valuable insights for further clinical investigations into the application of tislelizumab adjuvant therapy in the treatment of postoperative urothelial carcinoma.

### Supplementary Information

**Supplementary material 1.**

**Supplementary material 2.**

## Data Availability

The datasets used and analysed during the current study available from the corresponding author on reasonable request.

## Abbreviations

UC: Urothelial carcinoma
UTUC: Upper tract urothelial carcinoma
PD-1: Programmed cell death protein-1
PD-L1: Programmed cell death ligand-1
NLR: Neutrophil-to-lymphocyte ratio
PLR: Platelet-to-lymphocyte ratio
MLR: Monocyte-to-lymphocyte ratio
irAEs: Immune-related adverse events
DFS: Disease-free survival
OS: Overall survival
OR: Odds ratio
CI: Confidence interval
pN0: no lymph node involvement
pN +: lymph node involvement
