# Supplementary material for: Inflammatory markers predict survival in patients with postoperative urothelial carcinoma receiving tislelizumab (PD-1 inhibitor) adjuvant therapy
Source: BMC Cancer. 2024 Feb 12;24:196. doi: 10.1186/s12885-024-11969-5 (PMC10860305; doi:10.1186/s12885-024-11969-5)
Supplement: Supplementary file 2 — Supplementary material 2. [file 12885_2024_11969_MOESM2_ESM.docx]

We introduced statistically significant variables from univariate Cox regression (table 2) into multivariate Cox regression equation h(t,x)=h0(t)exp(3.012Z1-1.265Z2-0.812Z3), with Z1, Z2 and Z3 indicating pT stage, NLR and irAEs, respectively. Similarly, statistically significant variables from univariate Cox regression (table 3) were introduced into multivariate Cox regression equation h(t,x)=h0(t)exp(1.217Z1-1.578Z2-1.371Z3), with Z1, Z2 and Z3 indicating pT stage, NLR and irAEs, respectively.
